# Rac1b negatively regulates TGF-β1-induced cell motility in pancreatic ductal epithelial cells by suppressing Smad signalling

**DOI:** 10.18632/oncotarget.1696

**Published:** 2013-12-23

**Authors:** Hendrik Ungefroren, Susanne Sebens, Klaudia Giehl, Ole Helm, Stephanie Groth, Fred Fändrich, Christoph Röcken, Bence Sipos, Hendrik Lehnert, Frank Gieseler

**Affiliations:** ^1^ First Department of Medicine, University Hospital Schleswig-Holstein (UKSH), Campus Lübeck, Lübeck, Germany; ^2^ Clinic for Applied Cellular Medicine, University Hospital Schleswig-Holstein (UKSH) Campus Kiel, Kiel, Germany; ^3^ Institute for Experimental Medicine, Group Inflammatory Carcinogenesis, University Hospital Schleswig-Holstein (UKSH) Campus Kiel, Kiel, Germany; ^4^ Molecular Oncology of Solid Tumors, Internal Medicine V, Justus-Liebig-University Giessen, Giessen, Germany; ^5^ Institute of Pathology, University Hospital Schleswig-Holstein (UKSH) Campus Kiel, Kiel, Germany; ^6^ Institute of Pathology, Institute of General Pathology and Neuropathology, University Hospital Tübingen, Tübingen, Germany; ^7^ Current address: Department of Dermatology, UKSH, Campus Lübeck, Lübeck, Germany

**Keywords:** Rac1b, Rac1, Smad, TGF-β1, pancreatic ductal adenocarcinoma, cell migration

## Abstract

Transforming growth factor (TGF)-β1 promotes progression of pancreatic ductal adenocarcinoma (PDAC) by enhancing epithelial-mesenchymal transition, cell migration/invasion, and metastasis, in part by cooperating with the small GTPase Rac1. Prompted by the observation of higher expression of Rac1b, an alternatively spliced Rac1 isoform, in pancreatic ductal epithelial cells and in patients with chronic pancreatitis vs. PDAC, as well as in long-time vs. short-time survivors among PDAC patients, we asked whether Rac1b might negatively affect TGF-β1 prometastatic function. Interestingly, the non-malignant pancreatic ductal epithelial cell line H6c7 exhibited a higher ratio of active Rac1b to total Rac1b than the TGF-β1-responsive PDAC cell lines Panc-1 and Colo357. Notably, siRNA-mediated silencing of Rac1b increased TGF-β1/Smad-dependent migratory activities in H6c7, Colo357, and Panc-1 cells, while ectopic overexpression of Rac1b in Panc-1 cells attenuated TGF-β1-induced cell motility. Depletion of Rac1b in Panc-1 cells enhanced TGF-β1/Smad-dependent expression of promoter-reporter genes and of the endogenous Slug gene. Moreover, Rac1b depletion resulted in a higher and more sustained C-terminal phosphorylation of Smad3 and Smad2, suggesting that Rac1b is involved in Smad2/3 dephosphorylation/inactivation. Since pharmacologic or siRNA-mediated inhibition of Smad3 but not Smad2 was able to alleviate the Rac1b siRNA effect on TGF-β1-induced cell migration, our results suggests that Rac1b inhibits TGF-β1-induced cell motility in pancreatic ductal epithelial cells by blocking the function of Smad3. Moreover, Rac1b may act as an endogenous inhibitor of Rac1 in TGF-β1-mediated migration and possibly metastasis. Hence, it could be exploited for diagnostic/prognostic purposes or even therapeutically in late-stage PDAC as an antimetastatic agent.

## INTRODUCTION

Invasive pancreatic ductal adenocarcinoma (PDAC) is a fatal disease, for which no cure is currently available. The tumourigenic process involves the development of pancreatic intraepithelial neoplasia (PanIN), a common precursor lesion to PDAC also found in chronic pancreatitis (CP) which, in turn, is a well known risk factor for PDAC development [1]. The progression of PanIN lesions to carcinoma-in-situ is associated with oncogenic mutations in genes such as *K-ras*, *p53*, and *DPC4* in the ductal cells, resulting in deregulated cellular signalling [2]. Only four cellular signalling pathways have been identified that are genetically altered in 100% of pancreatic tumours [3]. One of these is the TGF-β signalling pathway comprising essentially two receptors with serine/threonine kinase activity (type II and type I/ ALK5) and the canonical Smad pathway. Signalling by Smad transcription factors is initiated by phosphorylation of Smad2 and Smad3 by the ALK5 kinase. Phosphorylated Smad2/3 subsequently forms a complex with Smad4, encoded by *DPC4*, which is translocated to the nucleus to control the transcriptional activity of TGF-β-responsive target genes [4]. In normal epithelial cells and early stages of carcinoma TGF-β/Smad signalling mediates growth inhibition and thereby inhibits tumour growth. However, as a consequence of mutations in *DPC4* and/ or hyperactivation of non-Smad pathways TGF-β can loose its tumour-suppressive function and in later stages of tumour development can become a potent tumour promoter [5]. Significant progress has been made in using transgenic mouse models for understanding the molecular mechanisms of how TGF-β signalling contributes to tumourigenesis of PDAC [6, 7]. These studies have shown that aggressive PDAC is caused by pancreas-specific blockade of TGF-β signalling in cooperation with active K-ras expression [7]. A recent study suggests that TGF-β/*K-ras*-mediated tumourigenesis is crucially mediated by the monomeric GTPase Rac1, since deletion of *Rac1* from the pancreas in a *K-ras*^G12D^ murine model prevented the formation of K-ras-driven tumours and significantly prolonged survival [8].

Rac1 is an ubiquitously expressed member of the Rho GTP-binding proteins that play key roles in cell morphology, motility, and mitosis [9]. It has been implicated as a modulator of oncogenic and metastatic signalling pathways [10, 11] and can be activated by hyperactive or overexpressed tyrosine-kinase receptors and active Ras. Rac1b is an alternatively spliced Rac1 isoform that differs from Rac1 by a 19 amino acid in frame insertion termed exon 3b behind the switch II region [12, 13]. Compared with Rac1, Rac1b has an accelerated GDP/GTP-exchange and an impaired GTP-hydrolysis, accounting for a self-activating GTPase [14, 15]. Although a few reports suggest protumourigenic functions for Rac1b, little is known on its specific role of initiation and progression of tumours. Thus, in colon cancer cells and serum-starved fibroblasts it drives cell cycle progression and sustains survival [16, 17] and in mammary epithelial/ breast tumour cells it enhances malignant transformation, genomic instability, MMP-3-induced epithelial-to-mesenchymal transition (EMT) [18], and cell motility/ spreading [19]. Unlike Rac1, Rac1b is unable to interact with Rho-GDI, to induce lamellipodia formation, to bind and activate p21-activated kinase (PAK) or the downstream kinase JNK [20], but has retained potential to activate NFκB [16] and to increase cellular reactive oxygen species [18].

Despite their functional cooperation in PDAC development, little information is available on a possible crosstalk between the Rac1/Rac1b and the TGF-β signalling pathways in the control of oncogenic cellular responses. We have shown earlier that Rac1 antagonized TGF-β1-induced growth inhibition and promoted cell migration in PDAC cells [21]. However, in studying Rac1-induced signalling and function one has to bear in mind that all Rac1 siRNA-mediated silencing approaches also result in co-depletion of Rac1b mRNA transcripts. Thus, most of these studies [8, 21] did not discriminate between the effects of Rac1 and Rac1b. In the light of our recent discovery of Rac1b expression in PDAC cells, a reinvestigation of the distinct roles played by Rac1 and Rac1b in the regulation of TGF-β1-induced cell motility appeared therefore mandatory. In contrast to Rac1, the role of Rac1b in these processes is unclear at present. Although it was reported that Rac1b promotes cell motility induced by MMP-3 [18], another study demonstrated that activated Rac1b was unable to stimulate lamellipodia formation [20]. Here we have used Rac1b-specifc RNA interference and ectopic overexpression of Rac1b in non-malignant and malignant pancreatic ductal epithelial cells. The non-malignant pancreatic ductal epithelial cell line H6c7 as well as the PDAC cell lines Panc-1 and Colo357 were particularly suitable because i) their TGF-β1-responsiveness is well characterized, ii) all cell lines respond to TGF-β1 with cell migration *in vitro* [21, 22] and iii) they were frequently employed in animal models for assessing the therapeutic activities of TGF-β inhibitors for suppressing pancreatic cancer growth and metastasis [23-25].

## RESULTS

### Rac1b is expressed in pancreatic ductal structures in chronic pancreatitis and PDAC

**Figure 1:**
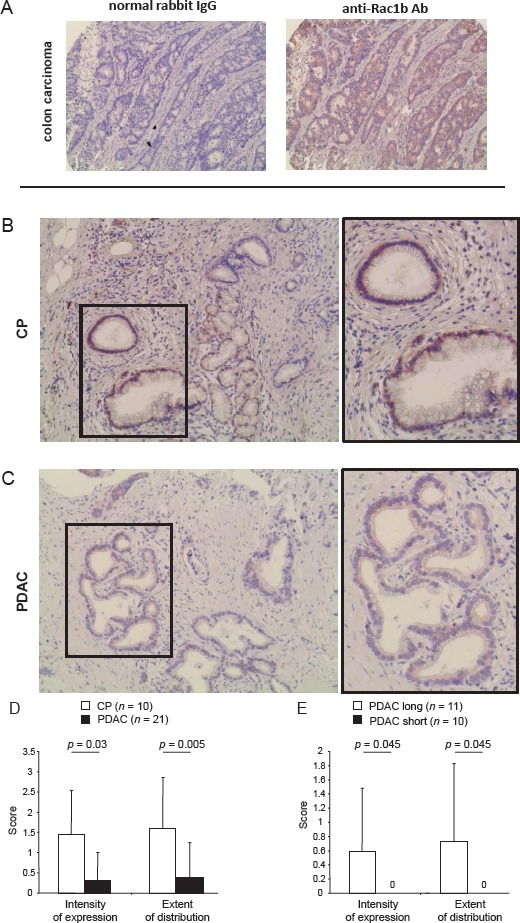
Immunohistochemical detection and quantification of Rac1b expression in CP and PDAC A, staining of colon adenocarcinoma with control rabbit IgG or specific anti-Rac1b antibody. Rac1b is expressed in pancreatic ductal structures in B, CP tissues and C, PDAC tissues. Images in frame in B and C represent enlargements of the indicated sections. Representative images are shown. Magnification in A, x 200, B+C, x 200, and x 400 for images in frames. D, Rac1b expression in ductal epithelia is higher in CP than in PDAC tissue and E, correlates with survival of PDAC patients. CP and PDAC tissues from the indicated number of patients (n) were scored for the staining intensity, refecting the intensity of expression and the extent of distribution given as % positivity of the whole section. Scoring systems are outlined in detail in “Materials and Methods”. Numbers above the graphs indicate statistical significance calculated by Shapiro-Wilk and Mann-Whitney-U-tests.

In order to evaluate whether Rac1b is expressed in pancreatic ductal epithelial cells under different pathological conditions, pancreatic tissues from CP or PDAC patients were analyzed for Rac1b expression (see Supplementary Tables 1 and 2 for clinical parameters of patients). As demonstrated in Figure 1A, Rac1b staining was established using colon carcinoma tissue in which Rac1b expression has been already described by RT-PCR [12]. In pancreatic tissues, Rac1b expression was predominantly found in ductal epithelial cells but partially also in acinus cells and stromal cells (Figure 1B, C). Interestingly, Rac1b expression in pancreatic ductal structures was more pronounced in CP than in PDAC tissues. Thus, in 7/10 CP tissues the majority of pancreatic ductal structures showed moderate Rac1b expression (Supplementary Table 1, Figure 1B) whereas in only 4/21 PDAC tissues Rac1b expression was determined mostly at a weak expression level (Supplementary Table 2, Figure 1C). The calculated differences as outlined in Figure 1D were statistically significant for both the intensity of expression (CP: 1.450±1.090 *vs*. PDAC: 0.310±0.698, *p*=0.03) and the extent of distribution (CP: 1.600±1.265 *vs*. PDAC: 0.381±0.865, *p*=0.005). Notably, Rac1b expression was detected in 4/11 PDAC patients with prolonged survival times (>23 months), whereas all patients with short survival times (≤9 months) completely lacked Rac1b expression. Again, differences as outlined in Figure 1E were significant for intensity of expression (long: 0.591±0.889 *vs*. short: 0±0, *p*=0.045) and extent of distribution (long: 0.727±1.104 *vs*. short: 0±0, *p*=0.045). Overall, these data indicate that Rac1b expression might be lost during pancreatic tumourigenesis and that loss is correlated with decreased survival.

### Rac1b is expressed in an active GTP-bound form in pancreatic ductal epithelial cells

**Figure 2:**
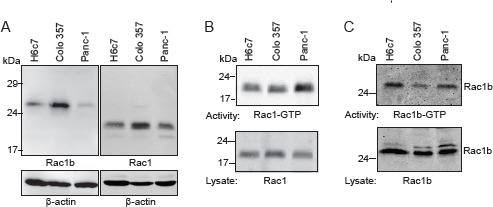
Rac1b is expressed in an active GTP-bound form in pancreatic ductal epithelial cells A, Rac1b and Rac1 expression was analyzed in 60 µg of cell lysates of H6c7, Colo357 (DPC4 wild type [26, 39]), and Panc-1 cells by immunoblot procedure and ECL detection using either a specific Rac1b polyclonal antibody or a Rac1 monoclonal antibody that recognizes both Rac1 and Rac1b (see also Figure 4A). Detection of β-actin served to control for equal loading. B, The amount of active Rac1-GTP was determined by affnity precipitation assays (upper panel) using 2000 µg of cell lysate and 30 µg of the Rac1-binding domain of PAK1B as an activation-specific probe to isolate GTP-bound Rac isoforms. To control for loading, aliquots of the sample (1/33) were analyzed in parallel immunoblots (lower panel). One representative assay out of ≥ three independent experiments is shown. C, The amount of active Rac1b-GTP was determined after adjustment of the amount of lysate to equal quantities of Rac1b. Therefore, 1500 µg of H6c7 cell lysate, 500 µg of Colo357 cell lysate and 2000 µg of Panc-1 cell lysate was used. Equal amounts of Rac1b were controlled by analyzing 1/20 of the samples in parallel immunoblots (lower panel) (n=3).

Having shown that Rac1b expression was higher in non-malignant ductal epithelial cells in CP than in cancerous tissues, we analyzed cell lysates of H6c7, Colo357, and Panc-1 cells for expression of Rac1b and Rac1 by immunoblot procedure using isoform-specific antibodies (Figure 2A). Due to the 19 amino acid in-frame exon 3b insertion in Rac1b, the protein migrates markedly slower than Rac1. Rac1b is highly expressed in Colo357 cells, less in H6c7 and only weakly in Panc-1 cells. In contrast, Rac1 is expressed mostly in Colo357 cells, followed by Panc-1 and H6c7 cells. The activity of endogenously expressed Rac1 and Rac1b was analyzed by using the Rac1-binding domain of PAK1B as an activation-specific probe to isolate GTP-bound Rac1 and Rac1b in affnity precipitation assays. The activity of Rac1 or Rac1b was assessed using cell lysates adjusted to equal amounts of Rac1 or Rac1b respectively, for better comparison. The activity of Rac1 varies with regard to the different cell lines. Panc-1 cells displayed a higher content of active Rac1-GTP than Colo357 and H6c7 (Figure 2B).

When the activity of Rac1b was assessed in cell lysates adjusted to equal amounts of Rac1b, meaning three times more lysate of H6c7 cells and four times more lysate of Panc-1 cells as compared to Colo357 cells, H6c7 cells still showed the highest amount of active Rac1b, followed by Panc-1 and Colo357 cells (Figure 2C). Densitometric analysis of the ratio of Rac1b-GTP/Rac1 normalized to the ratio of Colo357 cells led to the values: H6c7: 1.286±0.347, Colo357: 1.0, Panc-1: 1.236±0.139, n=6. However, given the only moderate differences in the activity/expression ratios, these results suggest that the amount of active Rac1b in a given cell is mainly regulated by the expression of the protein and to a minor extent by a modified activity.

### Selective depletion of Rac1b increases TGF-β1-mediated cell migration

**Figure 3:**
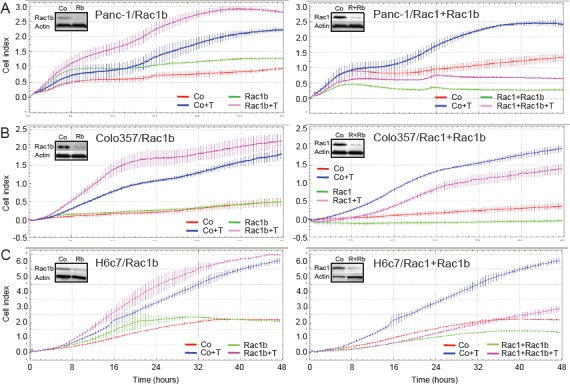
Effect of siRNA-mediated Rac1b knockdown on TGF-β1-induced cell motility in pancreatic epithelial cells A, Panc-1, B, Colo357 and C, H6c7 cells were transiently transfected twice with 50 nM siRNA to either Rac1b (Rac1b, left panel) or Rac1+Rac1b (right panel) and corresponding negative controls as indicated in the graphs and subjected to RCTA real-time cell migration assay using the xCELLigence DP system. Migration was performed in the absence or presence of TGF-β1 (T, 5 ng/ml) and measured for 48 h. Data of each condition and time point represent the mean ± s.d. of 4 wells. In each graph, differences between TGF-β1-treated control siRNA-transfected cells (blue curves) and TGF-β1-treated Rac1b siRNA or Rac1+Rac1b siRNA-transfected cells (pink curves) are highly significant (p<0.005, unpaired Student t test) at the 16, 24, and 32 h time points. Effective knockdown of Rac1b and Rac1 was verified by immunoblotting (inset, R=Rac1, Rb=Rac1b). Very similar results were obtained with the Rac1b/2 siRNA (not shown).

Next, we asked whether Rac1b also impacts on TGF-β1-induced cell motility. Using the RTCA assay with xCELLigence DP system in a chemokinesis setting, the effects of silencing Rac1b, and Rac1+Rac1b, on Panc-1 cell migration was analyzed (Figure 3). For this purpose, we initially employed two different siRNAs (Rac1b/1, Rac1b/2) that target the splice insertion sequence within Rac1b, a strategy that has been used in colorectal cancer cell lines before [17]. Using these siRNAs we were able to specifically knockdown Rac1b without affecting Rac1 levels. In some experiments, a Rac1 siRNA that targets both Rac1 and Rac1b (Rac1+Rac1b) [21] was transfected in parallel. The successful performance and specificity of these siRNAs was verified by qPCR and immunoblotting (Supplementary Figure 2).

TGF-β1 rapidly induced migration in control siRNA-transfected Panc-1, Colo357 and H6c7 cells with slightly different kinetics (Figure 3A-C). Transfection of Rac1b siRNA (Rac1b/1) strongly elevated TGF-β1-induced migratory activity in all three cell types (pink curves), as evidenced by the greater slopes of the curves (Figure 3A-C, left graphs). In contrast, transfection of Rac1+Rac1b siRNA strongly suppressed TGF-β1-mediated motility in Panc-1 and H6c7 cells (pink curves, Figure 3A and C, right graphs) in accordance with the promigratory role of Rac1 (Supplementary Figure 3, and Ref 21). At equal transfection efficiencies less dramatic but similar effects were seen in Colo357 cells (Figure 3B, right graph). The smaller effect compared to Panc-1 and H6c7 cells with regard to Rac1b was expected since Colo357 cells harbour lower levels of active Rac1b than Panc-1 and H6c7 cells (see Figure 2C). Together, these data clearly show that Rac1b, in contrast to Rac1, antagonizes TGF-β1-induced cell migration and that this inhibitory effect is seen in both malignant and non-malignant pancreatic ductal epithelial cells.

### Ectopic overexpression of Rac1b decreases TGF-β1-mediated cell migration

**Figure 4:**
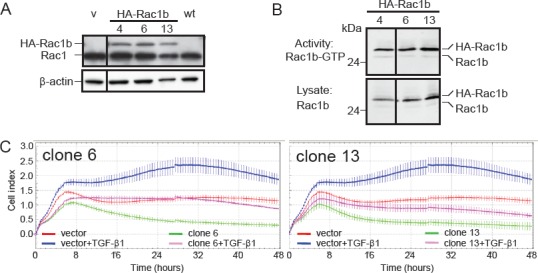
Effect of stable overexpression of Rac1b in Panc-1 cells on TGF-β1-mediated cell motility A, Immunoblot analysis of Panc-1 cells stably overexpressing hemagglutinin (HA)-tagged Rac1b encoded by pCGN plasmid. The Rac1 antibody used, which detects both Rac1 and Rac1b revealed two bands in the clones corresponding to Rac1 and HA-Rac1b. Empty vector-transfected (v) and wild type (wt) control cells lacked HA-Rac1b expression and also appeared negative for endogenous Rac1b due to the low expression level and underexposure of the blot. The vertical line between lanes 1 and 2 indicates that non-relevant lanes have been removed. B, Active HA-tagged Rac1b-GTP was determined by affinity precipitation assays (upper panel) using 1200 μg of cell lysate and 30 μg of the Rac1-binding domain of PAK1B. Aliquots of the sample (1/20) were analyzed in parallel immunoblots to control for loading (lower panel). HA-Rac1b and Rac1b were detected by using the Rac1b-specifc antibody. One representative assay out of ≥ three independent experiments is shown. C, Two Panc-1 clones overexpressing HA-Rac1b (clone 6 and 13) and vector control cells were analyzed for a total of 48 h in a RTCA assay for spontaneous and TGF-β1-induced (5 ng/ml) cell migration using a setting that measures chemokinesis as outlined in Materials and Methods. In both graphs, differences between TGF-β1-treated vector-transfected cells (blue curves) and TGF-β1-treated HA-Rac1b expressing cells (pink curves) are highly significant (p<0.001) at the 16, 24, and 32 h time points.

In order to confirm the inhibitory role of Rac1b in TGF-β1-induced chemokinesis, we generated cells with stable ectopic overexpression of HA-tagged Rac1b (Figure 4A). Panc-1 cells were chosen for this purpose because they exhibited low basal expression of Rac1b (see Figure 2). Figure 4B shows the expression and activity of HA-Rac1b (and endogenous Rac1b as an internal control) in three of these clones. All clones exhibit similar amounts of ectopically expressed HA-Rac1b with similar activity. This observation confirms the above notion that the amount of active Rac1b corresponds closely with expression of the protein. The same clones were subjected to RCTA chemokinesis assay under similar experimental conditions as shown in Figure 3. Rac1b suppressed both basal and TGF-β1-evoked cell motility relative to empty vector control cells (Figure 4C, two of three clones are shown). These results were substantiated and clonal artefacts ruled out by repetition of these assays with Panc-1 cells transiently transfected with expression vectors for either Rac1b or dominant-negative Rac1 (Rac1-N17), the latter of which supposedly inhibits Rac1, but not Rac1b (Supplementary Figure 3). These data are in line with the siRNA experiments and clearly show that Rac1b suppresses basal as well as TGF-β1-induced cell migration.

### The promigratory effect of Rac1b depletion is alleviated upon codepletion of Smad3

**Figure 5:**
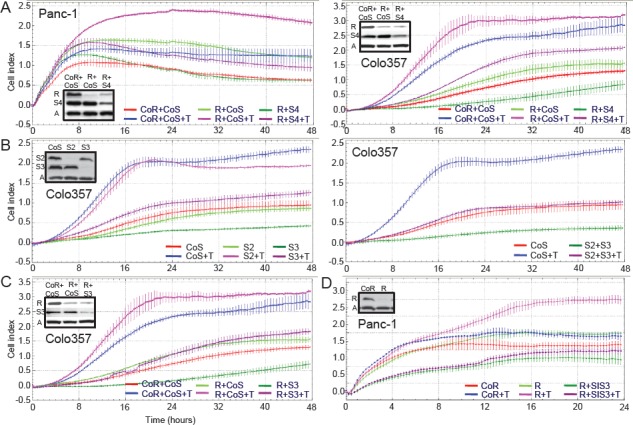
The promigratory effect of Rac1b depletion is alleviated upon codepletion of Smad3 A, Panc-1 and Colo357 cells were transiently cotransfected with 25 nM each of Co siRNA to Rac1b+Co siRNA to Smads (CoR+CoS), Rac1b siRNA+Co siRNA to Smads (R+CoS), or Rac1b siRNA+Smad4 siRNA (R+S4). Differences between TGF-β1-treated R+CoS-transfected cells (pink curves) and TGF-β1-treated R+S4-transfected cells (dark magenta curves) are highly significant (p<0.001) at the 16, 24, and 32 h time points. B, Colo357 cells were transiently transfected with 50 nM of Co siRNA (CoS), and either Smad2 siRNA (S2), Smad3 siRNA (S3) (left graph), or 25 nM each of Smad2 siRNA+Smad3 siRNA (S2+S3) (right graph). In both graphs, differences between TGF-β1-treated CoS-transfected cells (blue curves) and TGF-β1-treated S3-transfected cells (pink curves), or TGF-β1-treated S2+S3-transfected cells (dark magenta curves) are highly significant (p<0.001) at the 16, 24, and 32 h time points. C, Colo357 cells were cotransfected with 25 nM each of CoR+CoS, R+CoS, or R+S3. Differences between TGF-β1-treated R+CoS-transfected cells (pink curve) and TGF-β1-treated R+S3-transfected cells (dark magenta curve) are highly significant (p<0.001) at the 16, 24, and 32 h time points. D, Panc-1 cells were transiently transfected with 50 nM of either CoR or R. During TGF-β1 treatment one half of each transfection sample of the Rac1b siRNA transfectants was incubated with 10 µM of a chemical Smad3 inhibitor (SIS3). The Smad3-inhibitory effect of SIS3 was functionally confirmed in Panc-1 cells in a luciferase gene assay with the Smad3-responsive pCAGA-luc reporter (H.U., unpublished data). Differences between TGF-β1-treated R-transfected cells (pink curve) and TGF-β1+SIS3-treated R-transfected cells (dark magenta curve) are highly significant (p<0.001) at the 16, 24, and 32 h time points. All RTCA data represent the mean ± s.d. of 3 or 4 wells processed in parallel. Effective knockdown of Rac1b and Smad2, 3, and 4 was verified by immunoblotting (inset, A = β-actin). In A, C, and D very similar results were obtained with the Rac1b/2 siRNA (not shown).

TGF-β signalling through Smad4 has been shown previously to be crucial for TGF-β1-mediated cell migration in PDAC cells [26, 27]. We confirmed this by transiently transfecting Panc-1 and Colo357 cells with both a Rac1b siRNA (R) and a Smad4 siRNA (S4) and by analyzing TGF-β1-induced cell migration with the RTCA-system (Figure 5A). Indeed, Smad4 depletion dramatically reduced the Rac1b siRNA-induced promigratory TGF-β1 effect in both cell lines (Figure 5A). As depicted in the insets in Figure 5A, Rac1b depletion did not alter endogenous levels of Smad4. Next we determined whether the promigratory TGF-β1 effect of Rac1b depletion was mediated by Smad2 (S2) and/or Smad3 (S3) in PDAC cells using Colo357 cells for this purpose. We observed that only depletion of Smad3 but not Smad2 inhibited TGF-β1-induced cell migration and that simultaneous depletion of both Smads suppressed migration down to the level of untreated controls (Figure 5B). The Smad3 siRNA but not the control siRNA (CoS) also inhibited basal migration suggesting autocrine stimulation by TGF-β in culture. Together, these results indicate that TGF-β1-induced Colo357 cell migration is Smad3-dependent and suggests that Rac1b exerts its inhibitory function on cell migration primarily by suppressing the activation/function of Smad3. If so, the TGF-β1 effects resulting from Rac1b depletion, such as higher cell migration, should be alleviated upon Smad3 codepletion. To demonstrate this, we cotransfected cells with siRNA to Rac1b together with siRNA to Smad3. Interestingly, Smad3 inhibition by either siRNA (Figure 5C, left graph) or a chemical inhibitor (SIS3) (Figure 5C, right graph) alleviated the Rac1b depletion-specific increase in TGF-β1-induced increase in cell migration in Colo357 and Panc-1 cells, respectively. In conjunction with the data presented in Figure 4 we conclude that Rac1b inhibits TGF-β1-induced cell migration, probably through suppression of Smad3 activation and/or function.

### Rac1b negatively regulates TGF-β1 target gene expression and Smad-mediated transcription

**Figure 6:**
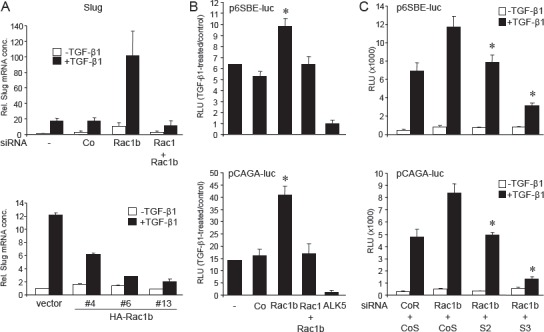
Rac1b negatively regulates TGF-β1-induced Slug expression and general Smad-mediated transcription A, upper graph, Effect of siRNA-mediated Rac1b and Rac1+Rac1b knockdown on TGF-β1-induced expression of Slug. Panc-1 cells were transiently transfected with transfection agent alone (-), 50 nM of control siRNA (Co), or 50 nM of siRNA to either Rac1b or Rac1+Rac1b and subjected to a 24 h TGF-β1 treatment followed by qPCR for Slug. Quantification of Slug by qPCR in three clones overexpressing HA-Rac1b relative to empty vector-transfected control cells. Note the reduced induction of Slug expression upon a 24 h treatment with TGF-β1. Data are from one representative experiment displayed as mean ± s.d. from 3 wells. B, Panc-1 cells were treated with transfection agent alone (-) or were transiently transfected with 50 nM each of Co, Rac1b, Rac1+Rac1b, or ALK5 siRNA. The next day cells were cotransfected with the same siRNAs together with pRL-TK-luc and either the Smad-specific reporter p6SBE-luc or pCAGA-luc as indicated. On day 3 cells were stimulated with TGF-β1 (5 ng/ml) for 24 h. Following lysis, firefly luciferase activity was measured and normalized for Renilla luciferase activity. Data are from one representative experiment (four experiments performed in total) representing the normalized mean ± s.d. Asterisk, p<0.001 compared to Co siRNA-transfected cells. C, As in B, except that Panc-1 cells were cotransfected with 25 nM each of Co siRNA to Rac1b+Co siRNA to Smads (CoR+CoS), Rac1b siRNA+Co siRNA to Smads (Rac1b+CoS), Rac1b siRNA+Smad2 siRNA (Rac1b+S2), or Rac1b siRNA+Smad3 siRNA (Rac1b+S3). Data are from one representative experiment (four experiments performed in total) representing the normalized mean ± s.d. Asterisks, p<0.001 compared to Rac1b+CoS-transfected cells. Effective knockdown of Rac1b, Rac1, ALK5, Smad2, and Smad3 was verified by immunoblotting (not shown).

Having established the antimigratory role of Rac1b for TGF-β1-induced cell motility, we asked whether Rac1b also interferes with regulation of migration/invasion-associated TGF-β target genes such as *SNAI2* encoding the protein Slug [28]. In Panc-1 cells, Slug is transcriptionally upregulated by TGF-β1 [29] in a Smad-dependent fashion [30]. Interestingly, Rac1b silencing rendered *SNAI2* hyperresponsive to TGF-β1 induction (Fig. 6A, upper graph), while its overexpression reduced induction of Slug expression upon a 24 h-incubation with TGF-β1 (Fig. 6A, lower graph). This data suggest that Rac1b normally antagonizes upregulation of Slug by suppressing TGF-β1 and, possibly, Smad3-mediated signalling.

To more directly study whether the inhibitory effect of Rac1b on TGF-β signalling is caused by inhibition of receptor-regulated Smads, we performed a series of luciferase reporter gene transfection assays using the Smad-responsive reporter plasmids p6SBE-luc (Smad2 and Smad3-responsive) or pCAGA-luc (primarily Smad3- responsive) and the Rac1b-specifc siRNA. The reporter gene assays revealed that silencing Rac1b strongly enhanced TGF-β1-induced reporter gene activity of both p6SBE-luc and, to a larger extent, pCAGA-luc (Figure 6B). In contrast, silencing Rac1+Rac1b had no significant effect relative to mock-transfected and control siRNA-transfected controls (Figure 6B), while siRNA to ALK5, used as positive control, potently abolished the sensitivity of both reporter genes to TGF-β1 stimulation (Figure 6B). In agreement with these results, transient expression of HA-Rac1b in Panc-1 cells decreased the sensitivity of pCAGA-luc to TGF-β1 treatment (Supplementary Figure S3).

Interestingly, cotransfection assays with Smad2 and Smad3-specifc siRNAs revealed that both Smad siRNAs were able to relieve the Rac1b siRNA-induced increase in TGF-β1-specifc reporter gene activity in p6SBE-luc and pCAGA-luc (Figure 6C). However, Smad3 depletion was much more effective than Smad2 depletion in this respect (Figure 6C). Very similar results were obtained with the Rac1b/2 siRNA (not shown). We conclude from these experiments that Rac1b primarily inhibits TGF-β1-induced Smad3-mediated transcriptional activity.

### Rac1b negatively regulates TGF-β1-induced Smad2/3 activation

**Figure 7:**
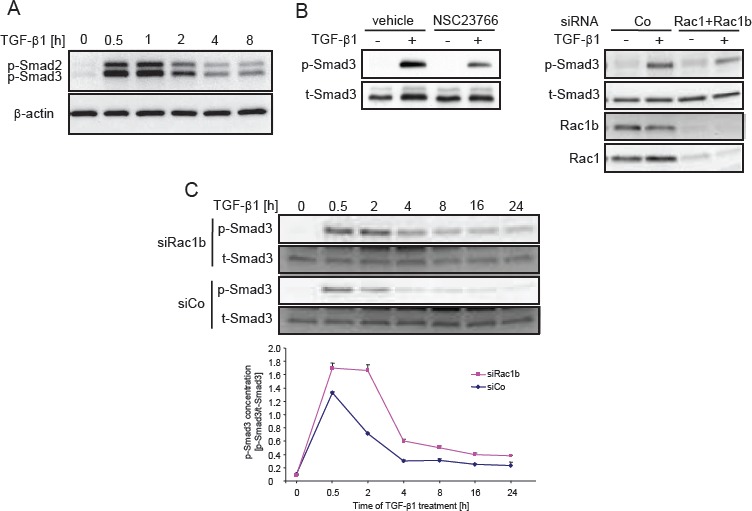
Rac1b negatively regulates Smad activation by TGF-β1 A, Kinetics of Smad2C and Smad3C phosphorylation in Panc-1 cells. B, Inhibition of Smad3C phosphorylation by the Rac1 inhibitor NSC23766 (left panel) or by transfection of Rac1+Rac1b siRNA (right panel). Successful inhibition of Rac1b and Rac1 expression was verified by immunoblot analysis with the respective antibodies. C, Kinetics of Smad3C phosphorylation in Panc-1 cells transfected with control siRNA (siCo) or Rac1b siRNA (siRac1b). Phosphorylated Smad3C was determined by immunoblotting (upper panel) and by a phospho-Smad3C-specifc ELISA (lower panel). Data represent the mean ± s.d. of triplicate samples. The ELISA was repeated two times with very similar results and one representative experiment is shown. p-Smad, phospho-Smad; t-Smad, total-Smad.

Activation of Smad2 and Smad3 proceeds primarily by the ALK5 kinase through phosphorylation of the two C-terminal Ser residues in the Ser-Xaa-Ser motif of the MH2 domain (Smad2/3C). In Panc-1 cells, phosphorylation of Smad2C and Smad3C in response to TGF-β1 is rapid with peak levels being reached at 0.5-1 h. Subsequently, phospho-Smad3C levels drop and approach baseline levels within 24 h (Figure 7A+C). We have shown previously that efficient phosphorylation of Smad2C by TGF-β1 in PDAC cells was abolished upon inhibition of Rac1 activity using Rac1-N17 [21]. In agreement with this finding, the generation of phospho-Smad3C was inhibited by either the chemical Rac1 inhibitor NSC23766 or Rac1+Rac1b siRNA in Panc-1 (Figure 7B) and H6c7 cells (data not shown). Given the dependence of TGF-β1-induced cell motility on Smad3 in conjunction with negative regulation of Smad3 transcriptional activity by Rac1b (see above), we speculated that Rac1b should decrease rather than increase Smad3C phosphorylation. The kinetic of Smad3C phosphorylation in Panc-1 cells transfected with siRac1b or control siRNAs was evaluated by both immunoblotting and ELISA (Figure 7C). Since cell motility is a long-term cellular response, we also included extended periods of TGF-β1 stimulation in this time course. To this end, TGF-β1-induced phosphorylation of Smad3C was significantly elevated in Rac1b-depleted cells as compared to control siRNA-transfected cells at all time points analyzed (Figure 7C). A similar time course was observed for phospho-Smad2C in Rac1b siRNA-transfected cells (data not shown). These results strongly suggest that Rac1b is involved in suppressing the Smad2/3C phosphorylation response. Moreover, this observation provides a mechanistic explanation for negative regulation of TGF-β1-induced transcriptional activity, gene responses, and cell migration by Rac1b.

## DISCUSSION

In this manuscript we demonstrate, for the first time, expression of Rac1b in tissues of patients with PDAC and CP. In both cases Rac1b expression was clearly localized to pancreatic ductal epithelial cells. *In vitro* experiments with ductal pancreatic cells of malignant and non-malignant origin revealed a hitherto unknown and quite unexpected functional role of Rac1b as a potent inhibitor of TGF-β-driven cell migration. In this respect, Rac1b acted as a natural antagonist of Rac1 which promoted TGF-β1-induced cell migration in these cells. Interestingly, an antagonistic relationship between Rac1 and Rac1b was also observed in HeLa cells following ectopic expression of Rac1b [31]. Rac1b interfered with Rac1 activation by PDGF, led to a reduction in membrane-bound Rac1, and promoted an increase in Rho activity which finally resulted in tumour-linked alterations in cell morphology and motility [31].

We disclosed here that Rac1b impacted on the TGF-β1/Smad3 signalling pathway in order to interfere with TGF-β1-induced cell migration. Cotransfection assays with Smad3-specifc siRNAs revealed that depletion of Smad3 was able to relieve the Rac1b siRNA-specific effect on cell motility. Since the reporter gene assays also showed that Rac1b silencing increased general Smad-mediated transcription, the question arose as to whether Rac1b is a more general regulator of Smad signalling. Although not in the focus of the current study, evidence was presented that other Smad3-mediated cellular responses, such as EMT, were inhibited by Rac1b as suggested by the finding that depletion of Rac1b potentiated TGF-β1-dependent upregulation of *SNAI2* (Figure 6A). Moreover, we found in H6c7 and Panc-1 cells that Rac1b antagonizes the TGF-β1 cytostatic effect (H. U., unpublished observation) suggesting that this potential prooncogenic function of Rac1b is already operating in pre-malignant pancreatic ductal epithelial cells.

The observation that Rac1b depletion prolonged TGF-β1-induced Smad3C phosphorylation (Figure 7C) and enhanced Smad3-specifc transcriptional activity of TGF-β1/Smad-responsive reporter genes and endogenous target genes such as *SNAI2* (see Figure 6) provides a molecular explanation for the large increase in TGF-β1-induced migratory activity following Rac1b depletion. In principle, Rac1b can target one of two steps to decrease phospho-Smad3C levels: It can either inhibit the ALK5 kinase activity and/or activate Smad-dephosphorylating phosphatases. In support of the first possibility are the observations that phosphorylation of Smad2 was affected in a very similar way (this study) and that ALK5 was detectable in Rac1 pulldown assays from MCF-10A cells overexpressing HER2 [32]. Given the fact that Rac1b is constitutively active and in this state is presumed to be located predominantly at the inner membrane, it may have a higher probability to interact directly or indirectly with the L45 loop of ALK5. Yet another possibility is that Rac1b is involved in TGF-β receptor endocytosis. Recently, it has been shown, that endocytosis of ALK5 has a role in ALK5-induced Smad signal termination rather than TGF-β signalling. Hence, by promoting endocytosis, Rac1b may inhibit ALK5-mediated Smad2/3 phosphorylation. Experiments are underway to solve this issue.

Another issue that is currently under investigation in our laboratory is the identity of the migration-regulating genes or pathways that are ultimately suppressed by Rac1b in either a Smad3-dependent or Smad3-independent manner. Besides *SNAI2* (see above), we focus on p38 MAPK which is known to be involved in TGF-β1-mediated cell migration [33]. We have preliminary evidence to suggest that Rac1b suppresses TGF-β1-induced activation of p38 MAPK and TGF-β1/ p38 MAPK-dependent cell migration (H.U., unpublished observation). Through such Smad-independent pathways a loss of Rac1b may allow for enhanced migration and invasion under the influence of TGF-β1 even in Smad4-negative pancreatic tumours.

So far, Rac1b expression was considered as being tumour-associated [12, 13, 34]. But only one study performed in human lung tumours provided protein data using western blot analysis and indications for a protumourigenic function. In this study, elevated Rac1b expression in tumour samples correlated with activating mutations in *K-ras* [34], but the cellular origin of Rac1b protein has not been determined. We are first to detect Rac1b by immunohistochemistry, showing considerable expression in ductal structures in pancreatic tissues from CP patients and, accordingly, in non-malignant H6c7 ductal epithelial cells. Indeed, Rac1b expression was found to be even lower in PDAC samples than in CP tissues. Notably, increased expression of Rac1b has also been described in patients with inflamed human colonic mucosa as well as following experimentally induced colitis in mice [35]. Data on upregulation of Rac1b in colon cancer [12] and the recent demonstration of Rac1b promoting *K-ras*-induced growth of lung adenocarcinoma [34] suggest an oncogenic role for Rac1b. Neither of these studies, however, have evaluated late phase of tumour progression characterized by tumour cell dissemination/metastasis in a TGF-β-rich tumour microenvironment. Based on our observations we hypothesize that tumour cells under such conditions are forced to carefully balance Rac1b to Rac1 expression and activity. High Rac1b expression/activity combined with a high ratio of Rac1b:Rac1 expression as in H6c7 cells would be expected to inhibit the potential for TGF-β-dependent migration and possibly metastasis. Conversely, in case of low or absent Rac1b expression/ activity or a low ratio of Rac1b:Rac1 expression as in Panc-1 cells, cancer cells might experience an enhanced potential for Smad3-dependent or independent migration, invasion, and possibly metastasis. The latter scenario appears to occur particularly in later stages of tumour progression and in a TGF-β-rich tumour microenvironment which is a characteristic feature of PDAC and associated with poor prognosis [36]. Our observation that patients with Rac1b expression have prolonged survival times could be interpreted mechanistically in such a way that Rac1b prevents activation of Smad3 and thereby limits prooncogenic events such as EMT and possibly (lymph node) metastasis. The hypothesis that Rac1b acts as an antimigratory factor by antagonizing protumourigenic Rac1 might be tested in mouse models of PDAC development [8, 37] or in tumour tissues from patients with different/earlier disease stages since in this study only tumours with the stage T3N1M0 were analyzed. Remarkably, however, we observed a higher proportion of specific staining for Rac1b in tumour sections from longtime survivors (4/11) than in short-time survivors (0/10).

Data from our laboratory revealed that the inhibitory effect of Rac1b on TGF-β1-induced cell migration was not restricted to pancreatic epithelial cells, but was also noted in MDA-MB-468 breast carcinoma and PC-3 prostate carcinoma cells. This would indicate that the role of Rac1b as an antagonist of TGF-β-induced cell motility may represent a more general phenomenon that is of physiologic relevance in other tumour entities. Our data suggest that Rac1b expression in PDAC may be exploited for diagnostic/prognostic purposes. Moreover, altering the balance of Rac1 to Rac1b expression and/or function towards Rac1b could represent a novel therapeutic approach in suppressing metastatic disease in PDAC and probably other carcinomas.

## MATERIALS AND METHODS

### Antibodies and reagents

The following agents and primary antibodies were employed: TGF-β1 (used at a concentration of 5 ng/ ml, RELIATech, Wolfenbüttel, Germany), the Smad3 inhibitor SIS3 [38] (Merck, Darmstadt, Germany, cat.#566405), the Rac1 inhibitor NSC23766 (Merck, Darmstadt, Germany, cat.#553502), anti-Rac1b (Millipore, Billerica, MA, cat.#09-271), anti-Rac1 (BD Transduction Laboratories, Heidelberg, Germany, cat.#610650), anti-Smad2 (Epitomics, Burlingame, CA, cat.#1736-1), anti-phospho-Smad2(Ser465/467) (Cell Signaling Technology, Frankfurt/Main, Germany, cat.#3101), anti-Smad3 (Abcam, Cambridge, UK, cat.#ab40854), anti-phospho-Smad3(Ser423/425) (Cell Signaling Technology, cat.#9514), anti-Smad4 (Santa Cruz Biotechnology, cat.#7966), anti-β-actin (Sigma, Deisenhofen, Germany, cat.#A5441).

### Cell culture and generation of cells overexpressing Rac1b

Human pancreatic carcinoma Panc-1 and Colo357 cells both of which are *DPC4* wild type [26, 39] and are representative of the quasimesenchymal subtype of PDAC [40] were originally obtained from the ATCC (Manassas, VA). The immortalized nontumourigenic pancreatic ductal epithelial cell line H6c7 [22] was a kind gift of M.S. Tsao (Toronto, Canada). The three cell lines were maintained as described earlier [22, 29] and their genotype was last confirmed in March 2013 by Short Tandem Repeat Analysis. Panc-1 cells stably overexpressing hemagglutinin (HA)-tagged Rac1b from pCGN plasmid (kindly supplied by C. Der, Chapel Hill, NC) were generated by transfection using Lipofectamine Plus (Life Technologies, Darmstadt, Germany) followed by selection of stable clones with 50 µg/ml hygromycin B (Sigma). Control cells expressing empty vector were cultured as pools of genetically heterogenous clones.

### Immunohistochemistry

Pancreatic tissues from 21 PDAC and 10 CP patients obtained from surgical specimens according to a protocol approved by the ethics committees of the University Hospitals Kiel and Tübingen (Permission number 110/99 and 470/210BO1, respectively) were investigated. After deparaffinization of formalin-fixed tissue sections, heat-mediated antigen retrieval was performed in citrate buffer (pH 6.0) in a microwave (4 x 5' at 150 W). Incubation with a Rac1b-specifc antibody or normal rabbit IgG diluted at a concentration of 5 µg/ml was performed for 30' at room temperature (RT). After washing with PBS, sections were incubated with anti-rabbit EnVision-HRP (Dako, Hamburg, Germany) for 30' at RT, washed with PBS and incubated with AEC Substrate (Dako) for 10'. Sections were first stained in Mayer's hemalum (AppliChem, Darmstadt, Germany) for 5' and after washing in water for 15' were fixed with Kaiser's glycerine gelatine (Waldeck, Munster, Germany). Rac1b expression in ductal epithelium was evaluated in a blinded manner by scoring the staining intensity, refecting the strength of expression (score: 0=none, 1=weak; 2=moderate; 3=strong) and the extent of distribution given as % positivity (score: 0=none; 1=<10%, 2=10-50%, 3=50-80%, 4>90%) of the whole section.

### RNA isolation and quantitative RT-PCR (qPCR) analyses

Total RNA from Panc-1 cells was isolated with peqGOLD RNAPure (Peqlab, Erlangen, Germany). The general RT-PCR protocol was described in detail earlier [29]. The mRNA expression was quantified by real-time PCR on an I-Cycler (Bio-Rad, München, Germany) with I-Cycler software (Bio-Rad). Amplification and detection of Rac1+Rac1b, Rac1b, and Slug-specific transcripts was achieved as described previously [29] using SYBR green supermix (Bio-Rad). All values for mRNA concentrations were normalized to those for glyceraldehyde-3-phosphate dehydrogenase (GAPDH) and TATA box-binding protein (TBP) in the same sample to account for small differences in cDNA input. The following primers were used for detection of Rac1+Rac1b: 5'-ACCATGCAGGCCATCAAGTGTGTGG-3' and 5'-TTACAACAGCAGGCATTTTCTCTTC-3'. Measurement of Rac1b was performed with exon 3b-specifc primers: 5 '-GGAGAAACGTACGGTAAGGATATAACC-3' and 5'-GGCAATCGGCTTGTCTTTGCCC-3' (see Supplementary Figure 1). For primer sequences of Slug, GAPDH, and TBP see [29].

### Transient transfections of Rac1 and Rac1b siRNAs

Panc-1, Colo357, and H6c7 cells were transfected with either of two Rac1b siRNAs. Rac1b/1 (5'-ACGUACGGUAAGGAUAUAATT-3') and Rac1b/2 (5'-CGUACGGUAAGGAUAUAACTT-3') [17] both corresponding to the mRNA motif ACGTACGGTAAGGATATAAC in exon 3b, and a corresponding negative scrambled control siRNA (CoR). These oligonucleotides were provided as Stealth siRNAs by Life Technologies as was a set of three validated siRNAs targeting *TGFBR1* (Stealth Select RNAiTM, Cat.#1299003). As control for cell migration assays, we purchased a mix of 3 premade siRNAs targeting Rac1 (siGENOME SMARTpool reagent) along with a siCONTROL nontargeting siRNA (all from Dharmacon via Biomol, Hamburg, Germany). As expected, this mix effectively silenced Rac1 and Rac1b (Supplementary Figure 1). Pre-evaluated siRNAs to either Smad2, Smad3, or Smad4 along with a negative control siRNA (CoS) were purchased from Qiagen (Hilden, Germany, see Geismann et al. [22] for functional evaluation). SiRNAs were introduced into Panc-1, Colo357 and H6c7 cells by two rounds of transfection with Lipofectamine RNAiMAX (Life Technologies) on day 1 and 2 after seeding. On day 3, transfected cells were treated with TGF-β1 for 24 h and subjected to RTCA assays (see below). In every transfection, aliquots of cells were tested for successful inhibition of siRNA targets by immunoblotting.

### Reporter gene assays

The luciferase assays were carried out as described in detail earlier [21]. Briefly, cells were seeded in 96-well plates and cotransfected on day 1 with various siRNAs using Lipofectamine RNAiMAX as described above. 24 h later cells received Lipofectamine 2000 (Life Technologies) along with the same siRNAs, the *Renilla* luciferase encoding vector pRL-TK-luc (Promega, Heidelberg, Germany) and either p6SBE-luc (kindly provided by S. E. Kern, Baltimore, MD), or pCAGA-luc (kindly provided by S. Dooley, Mannheim, Germany). On day 3, cells were treated with TGF-β1 for 24 h and luciferase activities determined with the Dual Luciferase Assay System (Promega). In all reporter gene assays, the data were derived from 6 wells processed in parallel and normalized with *Renilla* luciferase activity.

### Immunoblot analyses and ELISAs

The procedure for SDS-PAGE and blotting was performed as described in detail earlier [39]. In addition to immunoblotting, phospho-Smad3C was detected with the PathScan Phospho-Smad3 (Ser423/425) Sandwich ELISA Kit (#12003) and values were normalized to those of total-Smad3 measured with the PathScan Total Smad3 Sandwich ELISA Kit (#12002, both from Cell Signaling Technology).

### Rac1/Rac1b activity assays

The amount of active Rac1-GTP and Rac1b-GTP was determined by affinity precipitation assays as described [41] using the Rac1-binding domain of PAK1B as an activation-specific probe to isolate GTP-bound Rac isoforms. To control for loading, aliquots of the samples (1/20) were analyzed in parallel immunoblots.

### Real-time cell migration assays

Wild type and Rac1b siRNA-transfected cells were subjected to cell migration assays using the RTCA DP instrument (Roche, Mannheim, Germany) according to the instruction manual and previous descriptions [42, 43]. The xCELLigence RTCA technology has been shown to provide an accurate platform for non-invasive detection of cell motility and to exhibit strong correlations with conventional methods of measuring cell migration [43]. Prior to assembly of the CIM-Plates 16, the underside of the upper compartment was coated with 30 µl of collagen I (400 µg/ml) and allowed to dry. The assembled CIM-Plates 16 were equilibrated in medium containing 1% FBS with or without TGF-β1 (5 ng/ml) for 1 h at which background measurement was performed. Then 60,000 overnight serum-starved cells were loaded per well into CIM-Plates 16 with or without TGF-β1 and allowed to migrate for 48 h in medium containing 1% FBS. Data acquisition was done at 15' intervals and analyzed with the RTCA software (version 1.2, Roche).

### Statistical analysis

Experimental data are presented as mean ± standard deviation (s.d.) and were analyzed by the unpaired Student *t* test. Immunohistochemical data on Rac1b expression in CP and PDAC patients were analyzed using the Shapiro-Wilk and Mann-Whitney-U-tests. Data were considered statistically significant at *p*≤0.05.

## CONCLUSIONS

Our data suggest that Rac1b expression in PDAC may be exploited for diagnostic/prognostic purposes. Moreover, altering the balance of Rac1 to Rac1b expression and/or function towards Rac1b could represent a novel therapeutic approach in suppressing metastatic progression in PDAC and probably other carcinomas.

## AUTHORS' CONTRIBUTIONS

HU, SS, KG, SG, and OH performed the experiments. CR and BS provided the human CP and PDAC tissues and clinical data, and FF and HL contributed to the interpretation and discussion of the results. Both HU and FG are the principal investigators and were involved in the conceptualization and discussion of the manuscript. HU, KG, and SS wrote the manuscript. All authors read and approved the final version of the manuscript.

### Disclosure of Potential Conflicts of Interest

No potential conflicts of interest were disclosed.

### Editorial note

Acceptance of this paper was based in part on peer-review, conduced by another journal, revisions and further internal peer-review by Oncotarget

## Supplementary Figures and Tables




