# Supplementary material for: Rac1b negatively regulates TGF-β1-induced cell motility in pancreatic ductal epithelial cells by suppressing Smad signalling
Source: Oncotarget. 2013 Dec 23;5(1):277–90. doi: 10.18632/oncotarget.1696 (PMC3960208; doi:10.18632/oncotarget.1696)
Supplement: Supplementary file 1 [file oncotarget-05-0277-s001.pdf]

Rac1b negatively regulates TGF-β1-induced cell motility in pancreatic ductal epithelial cells by suppressing Smad signaling – Ungefroren et al

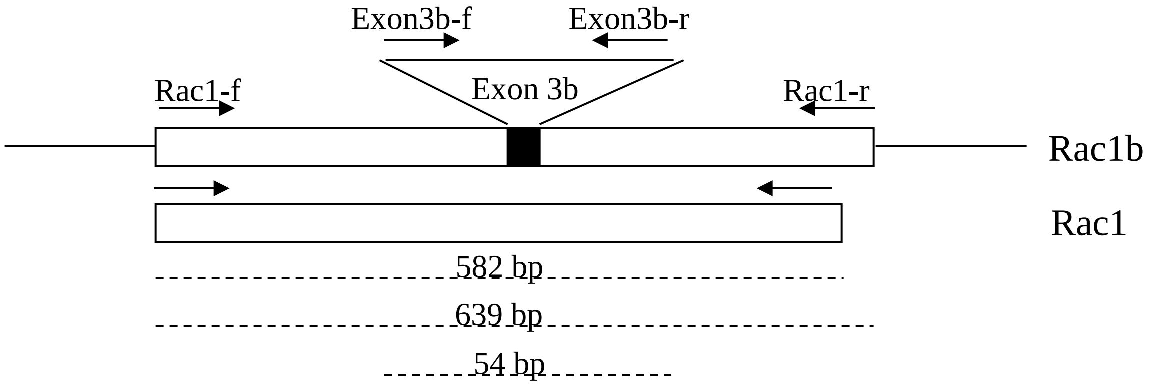

Figure S1: Schematic presentation of the Rac1b and Rac1 mRNA structure and localisation of primers for RT-PCR analysis. Note that Rac1b differs from Rac1 by the inclusion of an additional exon (exon 3b). A series of oligonucleotide primers was designed that specifically recognise either Rac1b (Exon3b-f+Exon3b-r) or both Rac1 and Rac1b (Rac1-f+Rac1-r). Numbers below the scheme indicate the length of the respective amplification products in base pairs (bp).

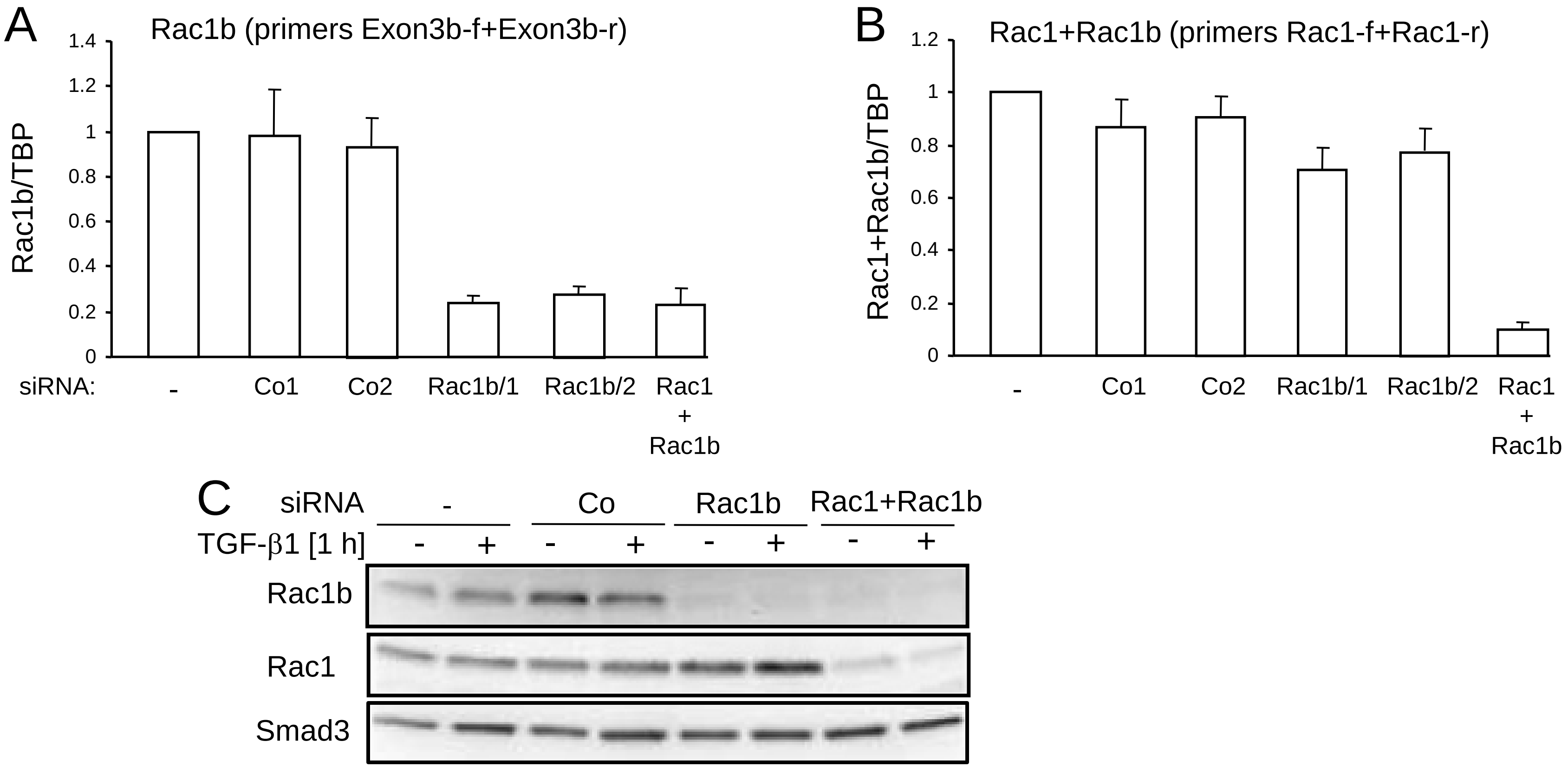

Figure S2: Evaluation of specificity of Rac1+Rac1b and Rac1b siRNAs. Panc-1 cells were transiently transfected twice on two consecutive days with Lipofectamine RNAiMAX alone (-), or Lipofectamine RNAiMAX along with 50 nM of siRNAs to either Rac1b/1 (ACG UAC GGU AAG GAU AUA ATT) or Rac1b/2 (CGUACGGUAAGGAUAUAACTT), a scrambled control siRNAs with identical GC content (Co1: ACG UGG CGG AAA UAA UUA ATT) or Rac1+Rac1b siRNA (siGENOME SMARTpool, a mix of 3 premade siRNAs) and corresponding control (Co2). Cells were harvested 24 h after the second round of transfection and analysed for Rac1b expression by qPCR analysis of (A) Rac1b using primers Exon3b-f and Exon3b-r and (B) Rac1+Rac1b using primers Rac1-f and Rac1-r (for location of primers see Supplementary Figure S1). A, strong knockdown of Rac1b was achieved in both Rac1+Rac1b-silenced and Rac1b-silenced cells, while transfection with a scrambled control siRNA, or medium with transfection agent alone, did not result in reduced levels of Rac1b mRNA. B, When using primers Rac1-f and Rac1-r (recognizing both Rac1 and Rac1b, specific detection of Rac1 was not possible due to the lack of unique sequences), we noted a 90% knockdown in Rac1 siRNA-transfected cells while only a slightly reduced signal was detected in Rac1b-silenced cells reflecting selective reduction of Rac1b. Data were normalized to the levels of TBP and represent the mean ± s.d. from three wells. C, The specificity of the employed siRNAs was confirmed by immunoblotting, probing the blots sequentially with Rac1b and Rac1. As expected, Rac1b siRNA suppressed endogenous Rac1b but not Rac1 protein levels while Rac1+Rac1b siRNA reduced both Rac1 and Rac1b protein levels. An antibody to total Smad3 was used as a loading control.

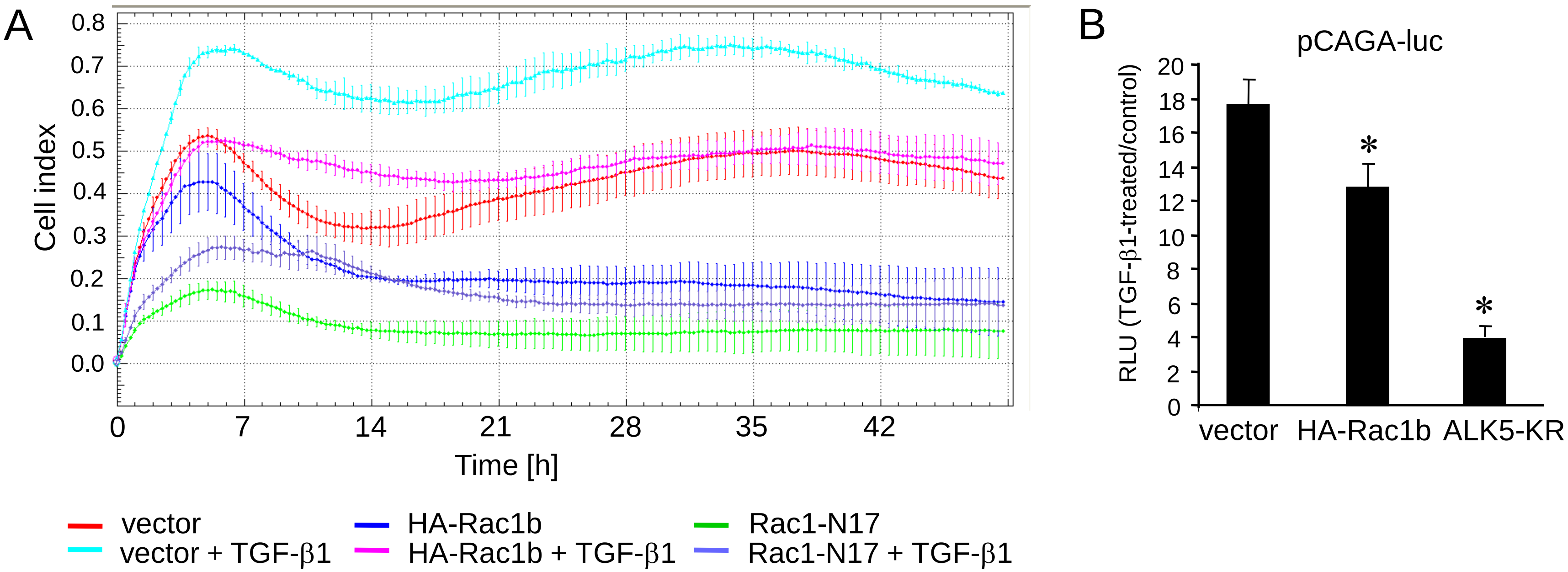

Figure S3: Effect of transient expression of HA-Rac1b on TGF-β1-induced migration (A) and general transcriptional activity (B) in Panc-1 cells. A, Panc-1 cells were transiently transfected with empty pcDNA3 vector, HA-Rac1b (obtained in pCGN and subcloned into pcDNA3), or dominant-negative Rac1 (Rac1-N17, in pcDNA3) using LipofectAmine 2000. Fourty-eight hours after the start of transfection, cells were stimulated, or not, with 5 ng/ml TGF-β1 and immediately analysed by RTCA assay for another 48 hours. Data represent the means ± s.d. of quadruplicate wells. One of two experiments both with very similar results is shown. B, Panc-1 cells were transiently transfected with empty pcDNA3 vector, HA-Rac1b, kinase-dead ALK5 (ALK5-K232R, in pcDNA3) along with pCAGA-luc and pRL-TK-luc using Lipofectamine 2000. Fourty-eight hours after the start of transfection, one half of the transfectants was stimulated with 5 ng/ml TGF-β1 for 24 hours and processed for dual luciferase measurement as described in Material and Methods. Data represent the mean ± s.d. of six wells. The asterisk indicates significance relative to the vector control. One of three experiments is shown, all with very similar results.
