# Supplementary material for: Rac1b negatively regulates TGF-β1-induced cell motility in pancreatic ductal epithelial cells by suppressing Smad signalling
Source: Oncotarget. 2013 Dec 23;5(1):277–90. doi: 10.18632/oncotarget.1696 (PMC3960208; doi:10.18632/oncotarget.1696)
Supplement: Supplementary file 2 [file oncotarget-05-0277-s002.pdf]

Rac1b negatively regulates TGF-β1-induced cell motility in pancreatic ductal epithelial cells by suppressing Smad signaling – Ungefroren et al

Supplementary Table S1. Rac1b expression in pancreatic ductal structures in CP patients and clinical parameter

No.	Intensity of expression	Extent of distribution	Gender male = 1 female = 2	Age
1	0	0	1	69
2	0	0	1	62
3	2	3	2	51
4	2	2	1	47
5	2-3	3	1	57
6	2-3	3	1	49
7	2-3	2	1	52
8	1	1	1	52
9	0	0	1	50
10	2	2	1	44

The following scoring systems were used: Intensity of expression: 0= none; 1= weak; 2= moderate; 3= strong; extent of distribution: 0= 0%; 1= < 10%; 2= 10-50%; 3= 50-90%; 4 > 90%.

Supplementary Table S2. Rac1b expression in pancreatic ductal structures in PDAC patients and clinical parameter

The following scoring systems were used: Intensity of expression: 0= none; 1= weak; 2= moderate; 3= strong; extent of distribution: 0= 0%; 1= < 10%; 2= 10-50%; 3= 50-90%; 4 > 90%. T, size of the primary tumor; N, regional lymph node metastasis; M, distant metastasis, refer to the TNM staging system for tumors. n.k, not known.

No.	Intensity of expression	Extent of distribution	Gender male = 1 female = 2	Age	T	N	M	Stage	Grade	Survival (month)
1	0	0	2	78	3	1	0	III	2	117
2	1-2	2	2	63	3	1	0	III	1	103
3	0	0	2	57	3	1	0	III	2	52
4	0	0	1	53	3	1	0	III	2	44
5	0	0	1	63	3	1	0	III	2	44
6	1	1	2	63	3	1	0	III	2	38
7	0	0	1	65	3	1	0	III	3	35
8	0	0	1	71	3	1	0	III	3	33
9	1-2	2	1	73	3	1	0	III	3	31
10	0	0	1	66	3	1	0	n.k.	2	30
11	2-3	3	1	62	3	1	0	III	2	23
12	0	0	2	75	3	1	0	III	2	9
13	0	0	1	46	3	1	0	III	3	8
14	0	0	2	73	3	1	0	III	3	6
15	0	0	1	62	3	1	0	III	3	6
16	0	0	2	76	3	1	0	III	2	6
17	0	0	2	61	3	1	0	III	3	5
18	0	0	1	59	3	1	0	III	3	1
19	0	0	1	65	3	1	0	III	2	1
20	0	0	2	70	3	1	0	III	2	1
21	0	0	1	75	3	1	0	III	2	1
